# On the Use of Electricity in Post-Partum Hæmorrhage

**Published:** 1874-02-01

**Authors:** Charles W. Earle

**Affiliations:** Chicago, Ill.


### MEDICAL EXAMINER.

A

*Semi-Monthly Journal of Medical Sciences.*

EDITED BY N. S. DAVIS, M.D., AND F. H. DAVIS, M.D.

No. III. CHICAGO, FEBRUARY 1ST, 1874. VOL. XV.

### Original Communications.

### ON THE USE OF ELECTRICITY IN POST-PARTUM HÆMORRHAGE.

BY CHAS. W. EARLE, M.D., CHICAGO, ILL.

MRS. M., an American lady, living on Sangamon street, in this city, summoned me, Dec. 9th, 1871, to attend her in confinement. I found her suffering from the usual cutting and annoying pains of the first stage, and in every respect in a fair condition, except a want of power in the pains.

On the morning of the 10th, Dr. Byford visited her (she having been under his treatment for uterine difficulty for some months previous to her pregnancy), and, excepting the uterine inertia spoken of above, expressed himself satisfied with her condition. He advised, however, if the labor was not concluded in a few hours, the use of forceps.

About 4 P.M., the uterus seeming powerless to complete the work, and, some twenty hours from the commencement of her labor, she was delivered, without any trouble, by the use of instruments.

As is my custom, a half drachm dose of fluid extract of ergot was administered, and, the after-birth complete was delivered in about fifteen minutes.

Without moving her from the position she was placed in for instrumental delivery, I sat down by the bedside to watch the condition of the uterus for one hour before putting on the binder and taking my departure. There had been such inertia of the womb during the entire labor that I was fearful of what my patient very soon experienced.

Without any premonition whatever, the uterus ceased its contraction, and a stream of blood, apparently as large as half my arm, came pouring from the vagina.

I immediately introduced my right hand to the fundus of the womb, and, with my left, tried to compress the descending aorta, giving orders at the same time to the attendants to administer more ergot, to lower the patient's head, apply cold water to the abdomen, and procure a piece of ice for inserting into the uterus. All this was done rapidly, and in much better order than is usual in such cases. But what a change there was in my patient! In two minutes she had changed from a most favorable condition—indeed, from a joyous and happy one—to an exsanguined, bloodless and pulseless state; apparently, she was moribund.

In addition to what I had already done, I gave what stimulants could be found in the house; and keeping my hands in the position noticed above, as the most effective way of stopping the largest amount of blood, sent immediately for Dr. I. N. Danforth, who lived in the immediate vicinity. He came forthwith, and, relieving me from my most fatiguing position, suggested port wine and carb. ammonia as the stimulant. Ergot had been given freely; ice, externally and internally, had been used; compression resorted to; stimulants and nourishing broths administered; but the hæmorrhage did not cease. Nothing, up to this time, had produced a good, strong continuous contraction of the uterus. Dr. Danforth now advised electricity; and in a very few minutes a battery was at hand; and placing one pole over the sacrum, and the other over the uterus, the current was commenced.

The effect was instantaneous and almost marvelous.

The uterus *contracted firmly;* the *hæmorrhage ceased immediately;* and as long as the electrical current was continued, the uterine tumor remained hard, and of proper size.

It was necessary, however, to keep up the current for some time; for, as soon as we ceased using the electricity, the womb softened, and blood commenced to flow. It was above twelve hours before we ceased using the instrument altogether. At that time the adynamic condition of the entire system, and uterus especially, seemed to be overcome, and we felt safe in leaving our patient.

The lady was saved, and made a very comfortable convalescence. Electricity certainly contributed largely to the favorable result.

As I am preparing these pages for publication, Dr. Danforth informs me that he has had a case in his own practice, in which electricity was supplied.

The indications for treatment were so marked, and the physiological application of electricity attended with such beautiful results, that I feel that my article will be made of double importance to the profession by its insertion.

He very kindly places the notes of the case at my disposal, which I give entire.

"October 13th, 1872, I was called upon to attend Mrs.——, a lithe, active, healthy brunette, about twenty-three years of age, in her first confinement. The patient was, and is, alike remarkable for her quick, active movements and powers of endurance, and for her slight physical proportions and fragile appearance. She passed through gestation with very little inconvenience, superintending her household affairs to the day before her labor, and maintaining her wonted cheerfulness and vivacity, not only *to* her sickness, but well *through* it. In view of the fact that my patient was so unusually small, I apprehended a long and probably tedious labor; but comforted myself with *another* fact, that I have many times observed, namely, that a brunette will endure a far more tedious labor, and retain her strength and courage much longer, than a blonde; hence, I felt warranted in hoping for a favorable termination. *

"Labor began about the middle of the afternoon of the twelfth—or, perhaps, it is more correct to say premonitions of labor, since nothing but "teasing" pains occurred till the evening was somewhat advanced. About nine o'clock in the evening, an examination disclosed a natural presentation, a very small amount of liquor amnii; and the os uteri dilated to the extent of about one inch; pulse natural, and the patient's condition quite satisfactory. About twelve o'clock, the os was fully dilated, and the head passed the upper strait, and shortly passed forward to the perinæum, which still remained quite rigid and unyielding. But as the pulse was yet perfectly normal, and the strength did not appear to flag, I saw no occasion for alarm. The pains continued with regularity, and everything progressed quite as well as I had anticipated, up to about four o'clock on the morning of the 13th. About this time, the patient began to show the evident results of a hard night's work, and indications of the not far distant exhaustion of her capital stock of strength, in spite of our efforts to sustain her by concentrated nourishment. The pulse had risen to 100; the patient looked weary; the temperature began to rise; and a nervous restlessness took the place of her former cheerfulness. But, as the perinæum was not yet in a favorable condition, I felt constrained to postpone delivery a while longer, although convinced that a forceps delivery would be necessary. At the end of another hour (about five o'clock) the patient's symptoms were as follows: Pulse 120; skin hot and dry (the actual temperature was not taken); complains of thirst; looks weary and restless, and begins to feel discouraged; occasionally draws a long sigh, to give expression to her exhaustion; and is "fidgety," nervous, and impatient. The vagina begins to feel hot and dry, but the parts are quite well dilated, and the head is within easy reach of the forceps. Believing it to be my duty to deliver without further delay, I at once applied the forceps, and accomplished the delivery of the child, a healthy boy, without accident, and with the expenditure of far less force than I expected. In a few minutes, the placenta was expelled naturally, the uterus contracted promptly; the usual bandage and compress was applied; the patient expressed herself as "feeling comfortable;" and I congratulated myself upon the fortunate issue of the case. After sitting awhile —perhaps twenty minutes—by the

* I think the majority of physicians will bear me out in the statement that women of dark complexion are far more likely to have longer, as well as more severe, labors, than those with light skins; also, that they generally make quicker and more perfect recoveries, and manifest greater resistance to septic influences. Did space permit, I could adduce many proofs of this.—I. N. D.

bedside, I had occasion to leave the room, and was absent, I imagine, for another twenty minutes. As I re-entered the room, the patient *gasped*, rather than *said*, "Doctor, how dark everything looks." Placing my left hand upon the abdomen, I felt the uterus distended and swollen, with an enormous coagulum, which I turned out by thrusting my right hand into the womb with all possible expedition. I then attempted to secure contraction by intra-uterine irritation with the fingers, and by "teasing" the organ through the abdominal wall. But, although the uterus would feebly contract, it would immediately relax again, and the loss of blood continued until my patient seemed upon the very verge of death. Meantime, I had sent for my battery, which was happily near at hand, and was, therefore, quickly at the bedside. I immediately applied one pole over the uterus, and the other over the spinal column. The result was simply magical; never in my professional experience have I seen aproaching death so promptly arrested, or felt such a burden of anxiety lifted from my shoulders, as it were, in a moment. Under the electric goad, the flabby and toneless uterus immediately became a hard, round ball, no larger than my two fists, and the bleeding ceased. Of course, the pillows were taken away, the foot of the bed was raised, and brandy, beef-tea, and milk, were alternately administered, as fast as I thought the stomach would retain it. *

"After the application of a gentle current, for ten or fifteen minutes, the poles of the battery were removed, and I sat down to watch the uterus, with my hand upon the abdomen. *Relaxation came on again after a very short time, and the organ commenced refilling with blood.* Several times more, in course of the succeeding three or four hours, I attempted to suspend the use of the battery, but with precisely the same results. It was well along in the afternoon of the 13th, before I dared leave my patient, or cease using the battery. In fact, I was obliged to "hold on" to the womb with electricity, until, by virtue of stimulants and beef-juice, it had acquired strength and tone enough to take care of itself. I am profoundly impressed with the conviction that I should have seen this patient die before my eyes, but for electricity. The pulse was gone; a mere "flicker" was perceptible to the ear over the heart; she was blanched, bloodless, and speechless; in fact, she was in profound collapse, with extreme uterine inertia; and the blood was still passively draining from the flaccid uterine sinuses, in spite of the vigorous application of the ordinary measures within my reach. At this point, the battery came, and the case assumed another aspect. Instead of standing helplessly by, resorting to futile expedients, I became at once master of the situation. For the electric current does more than merely to whip up the uterus, and make it contract; it gives a fillip to the whole nervous system, and arouses it to another effort in its own behalf; it causes the heart to contract more forcibly, and thus sends the starving brain a new supply of blood; it seems, in some sort, to fur-

* I think, in cases of severe hæmorrhage, with extreme exhaustion, the mistake is often made of pushing stimulants and nourishment too fast. The stomach itself shares in the general exhaustion, and cannot perform its duties as rapidly as usual. Hence, we are likely to have overloading and vomiting.—I. N. D.

nish a momentary "motive power" to the whole machine, thus giving the patient one more chance of life. Meantime, we are trying to restore the elements of the blood as fast as possible, by various forms of concentrated nourishment."

In conclusion, I will merely add that Mrs.—— made an excellent recovery, without accident or mishap of any kind.

In presenting the preceding cases of post-partum hæmorrhage to the profession, in which very marked beneficial results are claimed for electricity, I do not wish the reader to think for a moment that I wish to undervalue the powerful remedies which are ordinarily used with good success in these critical cases, or that I wish to give electricity undue prominence.

We all recognize the fact, that severe flooding after childbirth places our patient in a most perilous condition. It is an occurrence that demands all the nerve, skill and presence of mind which we possess.

We must not make any mistakes. We have no time to consult authorities. And none of us should dare to enter the lying-in chamber without a knowledge of every agent of service in post-partum hæmorrhage, and the power to promptly and intelligently use them.

To make this article as useful as possible, and to show that electricity has not been recognized by many of our prominent authors and teachers as an excito-motor stimulant of the highest power, I make the following extracts:

* Dr. Elliot expresses his conviction, that deaths from post-partum hæmorrhage rank among the most preventable causes of death, and regards the practitioner responsible for proper treatment. His practice, as recorded in his writings, seems to me to have been particularly good, and worthy to be followed. It is decidedly against hurrying away from the bedside after delivery, as I am afraid is the custom of many.

Ergot, the hand in the uterus, and cold, in case post-partum hæmorrhage takes place, and the retention of the hand over the fundus for some time after the delivery of the placenta, in *every* case, as a matter of safety, will be found recommended in his work. He does not speak of electricity.

Schroeder, in a very recent work,* does not mention any new theory, nor does he speak of electricity. He relies largely upon pressure, and gives ergot, but does not trust the drug absolutely.

Dr. Meadows † relies on stimulants, the hand in the uterus, the cold douche, ergot, and, lastly, transfusion. He does not mention electricity.

Prof. Byford ‡ speaks of post-partum hæmorrhage as one of the most fearful and appalling accidents which can befall a woman. "Atony," he remarks, "is the condition of the uterus in which it occurs."

Ergot, grasping and kneading the uterus, ice, or ice-water, to the abdomen, or in the uterine cavity, and compression with very solid substance, like a book, sufficiently firm to close the uterine cavity, are the means employed for combating the dangerous complication.

* Obstetric Clinic.

* Manual of Midwifery, 1873.
† Manual of Midwifery, 2d Edition.
‡ Theory and Practice of Obstetrics.

Cazeaux* recommends, in obstinate hæmorrhage, the tampon; the introduction of a bladder into the womb; the approximation of the uterine walls by immediate pressure; compression of the aorta; the use of ergot; of opium; and transfusion.

In regard to the tampon, it is urged by those who advise it that it is not so much to stop the blood, as it is an *irritant* to the internal surface of the womb. It *must not* be used in such a manner as to convert an external into an internal hæmorrhage.

Bedford,† after speaking of the usual remedies used, says: "Electricity, for example, has been much lauded by certain English authorities; but you must at once recognize a very serious objection, which is the delay necessarily connected with its application, simply for the reason that the apparatus is not at hand. Often, before it could be obtained, death will have claimed its victim."

Dr. Rigby ‡ speaks of an electrical machine to produce uterine contraction after the Cæsarean section.

While speaking of most of the remedies used in post-partum hæmorrhage, perhaps I should not fail to mention the method proposed by Dr. Barnes. The solution, according to this gentleman, should consist of the following:

℞.—Liq. ferri perchloridi fort, ℥ iv.

Aquæ, ℥ xii.

This should be thrown into the uterus, quite to the fundus, the operation being performed slowly and with care.

A weaker solution than the one mentioned above has been used, by competent men, and with good results.

I should say that this operation has its evil consequences; sometimes they are experienced, and, in other cases, they have not been noticed.

When present, they are: long continued and severe after-pains; tenderness in the lower part of the abdomen; fever; weakness; and the discharge, for several days, of small particles of the iron, with small clots.

It will be observed that electricity is mentioned but twice; and, in one instance, with what seems to the author a very serious objection. The objection, at this time, can hardly be urged; for electrical machines of some kind are in the possession of very many practitioners, and, in a majority of cases, can always be procured. We should *know*, at least, that, in some cases most marked results have been accomplished by their use.

The kind of instrument used in uterine inertia is important; a galvanic current would be very much less effective than the faradic current.

In the minds of many who have not had access to recent works published on electricity, the differential indications between the two currents are both erroneous and imperfect.

Many suppose that there is a marked difference in kind; indeed, that there are two different forces.

There is very strong evidence at this time, however, for regarding the two currents as different in *degree*, rather than *kind*.

Beard and Rockwell have produced about the same therapeutic results with one as with the other; yet each current has its advantages.

One advantage of the faradic over the galvanic, is its frequent interrupt-

* Theoretical and Practical Midwifery,

† Principles and Practice of Obstetrics, p. 396.

‡ Obstetric Memoranda.

ions. This current should be used in uterine inertia. When we wish to produce full muscular contraction, and in muscles which are not diseased, as we shall see presently, the faradic is the current indicated.

Beard and Rockwell give the following general differential indications for the use of the two currents:

"The galvanic should be used—

"1st, To act with SPECIAL ELECTROLYTIC POWER on the brain, spinal cord, sympathetic, or any part of the central or peripheral nervous system;

"2d, To produce contractions in paralyzed muscles that fail to respond to the faradic.

"3d, In electro-surgery, to produce electrolysis or cauterization.

"The faradic should be used—

"1st, To act MILDLY on the brain, spinal cord, sympathetic, or any part of the central or peripheral nervous system;

"2d, To excite muscular contractions wherever the muscles are not so much diseased as to be unable to respond to it;

"3d, To produce strong mechanical effects." *

The practical points which I wish to bring out in this article, are the following:

1. Post-partum hæmorrhage being an exceedingly dangerous conclusion to labor, and liable to take place when we least expect it, we should always be prepared to combat it. We should remain by the bedside of our patient at least one hour after delivery, and should repeatedly satisfy ourselves that the uterus has firmly contracted.

2. Every practitioner should be perfectly familiar with the the ordinary methods of treating post-partum hæmorrhage; *he should have ergot with him at confinements*, and, when it is possible, should have ice at hand, in readiness for use.

3. From the known physiological action of electricity, and clinical observation of a few recorded cases, the indications for treatment in uterine inertia are best met, and most safely combated, by the use of the faradic current.

* For the above rules, see a valuable work "On the Medical and Surgical Uses of Electricity," by Beard and Rockwell.

